# Myasthenia Gravis in Pregnancy: Prenatal and Postnatal Diagnostic Challenges—A Narrative Review

**DOI:** 10.3390/diagnostics16060899

**Published:** 2026-03-18

**Authors:** Angeliki Gerede, Maria Danavasi, Efthymios Oikonomou, Panayiota Papasozomenou, Vasiliki Kourti, Anastasios Potiris, Christos Chatzakis, Sofoklis Stavros, Nikoletta Koutlaki, Makarios Eleftheriadis

**Affiliations:** 1 Department of Obstetrics and Gynecology, Democritus University of Thrace, Alexandroupolis Campus, 69100 Alexandroupolis, Greece; mairidanavasi@gmail.com (M.D.); efthymiosoikonomou@gmail.com (E.O.); vasiliki.kourti29@gmail.com (V.K.); nkoutlak@med.duth.gr (N.K.); 2 School of Health Science, International Hellenic University, 57400 Thessaloniki, Greece; ppapasoz@ihu.gr; 3 Third Department of Obstetrics and Gynecology, University General Hospital “ATTIKON”, Medical School, National and Kapodistrian University of Athens, 12462 Athens, Greece; apotiris@med.uoa.gr (A.P.); sfstavrou@med.uoa.gr (S.S.); 4 Second Department of Obstetrics and Gynecology, “Aretaieion” University Hospital, Medical School, National and Kapodistrian University of Athens, 11528 Athens, Greece; cchatzakis@gmail.com

**Keywords:** myasthenia gravis (MG), autoimmune disease, immunological biomarkers, perinatal outcome

## Abstract

Myasthenia gravis (MG) is a prevalent autoimmune disorder affecting neuromuscular junctions, typically characterized by muscle weakness due to autoantibodies targeting acetylcholine receptors (AChR) or muscle-specific kinase (MuSK). Generalized MG is a more severe form of the condition than ocular MG. Although MG can strike at any age, young adult women are typically affected, especially in their reproductive years. MG is rare during pregnancy, with the first trimester and the postpartum period being the most common times for exacerbations. The influence of MG on pregnancy outcomes remains ambiguous, with some studies finding larger prevalence of issues such as preterm birth and small-for-gestational-age babies, while others indicate results similar to the general population. Management of MG during pregnancy necessitates careful monitoring and drug adjustments. Teratogenic concerns make several immunosuppressive drugs, such mycophenolate mofetil and methotrexate, contraindicated. In contrast, medications like prednisolone and pyridostigmine are generally recognized as safe. Women with MG may have flare-ups after giving birth, and infants may have transient neonatal myasthenia gravis. Comprehensive prenatal treatment and multidisciplinary assistance are crucial for promoting maternal and fetal health during pregnancy in women with MG. This paper examines the relevance of immunological biomarkers, RNAs, and other novel biomarkers in myasthenia gravis (MG). It emphasizes the need for more investigation to determine their role in the pathogenesis of MG, evaluate biomarker profiles across subgroups, and look at changes after treatment. The study also underlines the significance of high-throughput investigations to detect new biomarkers and reveal genetic variables impacting MG pathogenesis.

## 1. Introduction

Myasthenia gravis (MG) is the most frequent autoimmune disorder affecting the neuromuscular junction. The majority of affected individuals will develop antibodies targeting muscle-specific kinase or the acetylcholine receptor (AChR) [1]. This phenomenon leads to decreased nerve impulse transmission due to striated muscle fibers. The skeletal muscles—notably those involved in respiration, eye function, and leg movement—often demonstrate variable degrees of weakening in affected persons [2]. Although MG is frequently curable, it can cause serious consequences and even be lethal in certain situations [3]. This is often preventable with early discovery and effective treatment.

A difference is formed between a generalized type and an ocular form, with the latter having a more favorable prognosis. Although it is not inherited, multiple members of the same family may be diagnosed with it. Autoantibodies that target the nicotinic acetylcholine receptor or other postsynaptic antigens (muscle-specific tyrosine kinase, low-density lipoprotein receptor-related protein 4, agrin) at the neuromuscular junction cause a disruption in nerve impulse transmission to skeletal muscles, which leads to clinical manifestations [4]. Affected people commonly develop thymic hyperplasia.

According to Dresser et al. (2021), the symptoms range from a purely ocular presentation to a notable weakness of the limbs, bulbar, and respiratory muscles [3]. Myasthenia gravis is a disorder characterized by a variable progression, with occasional exacerbations that demand more severe intervention. People have increased muscle weakness or related symptoms during an exacerbation, and they may require higher dosages or additional drugs to control their symptoms. A myasthenic crisis is a life-threatening disorder characterized by weakness of the respiratory and/or bulbar muscles, resulting in breathing compromise that requires ventilatory assistance.

From a pathophysiological and phenotypic basis, myasthenia gravis is a diverse condition. Although it can happen at any age, including childhood, myasthenia gravis most usually affects young adult women (those under 40). The disorder generally has a bimodal age distribution, characterized by two incidence peaks; one in the third decade and another post-sixth decade, predominantly affecting females in the younger cohort [5]. Although being rare, with a reported rate of 0.3–7.7 per 100,000 in the general population, the incidence of MG increases for women over their second and third decades of life, potentially compromising pregnancy [1].

The diagnosis of myasthenia gravis (MG) is established via clinical and physical tests, with confirmation acquired utilizing serum immunoassays that detect autoantibody levels. Myasthenia gravis therapy generally comprises symptomatic care with anticholinesterase drugs, while steroids and other immunosuppressive medicines are applied in cases of more severe sickness. Intravenous immunoglobulin (IVIG) and/or plasmapheresis may be utilized for persistent cases or myasthenic crises. Thymectomy has been shown to alleviate symptoms in persons with thymoma or thymic hyperplasia in situations of early-onset AChR antibody positivity.

Myasthenia gravis during pregnancy is relatively rare, affecting around 1 in 30,000 pregnant women worldwide, but the prevalence fluctuates in various geographical regions [4]. Myasthenia gravis has an uneven progression throughout pregnancy. Pregnancies in moms with myasthenia gravis can result in adverse outcomes. Exacerbation is most likely to occur during the first trimester and the postpartum period, although it can happen at any time. Previous studies indicate overall pregnancy exacerbation rates of around 30%; however, both greater and lower rates have been documented [1]. As for pregnancy outcomes, the literature is similarly contradictory, with some studies reporting higher rates of small-for-gestational-age (SGA) babies, preterm birth (PTB), cesarean delivery, and preterm prelabor rupture of membranes (PPROM), while others report rates comparable to the general population [1]. AChR autoantibodies may infiltrate the fetal circulation by exploiting physiological transfer mechanisms, potentially impacting the fetal neuromuscular junction and resulting in a complication known as transient neonatal myasthenia gravis (TNMG), a self-limiting myasthenic syndrome that affects neonates [4]. TNMG is commonly said to be 10–15%, with independent series reporting rates between 3 and 4% and 33% [1].

## 2. Pathophysiology of MG in the Context of Pregnancy

MG’s classification as an IgG-antibody-mediated illness is closely related to its impact on pregnancy, labor, and fetal development. In myasthenia gravis, autoantibodies assault molecules in the postsynaptic membrane at the neuromuscular junction. These antibodies typically attach to the acetylcholine receptor (AChR) or associated molecules such as muscle-specific kinase (MuSK) or lipoprotein-related protein 4 (LRP4). Their binding results in a loss of both the activity and number of AChRs, largely via antibody-mediated complement activation and AChR cross-linking, which is recognized as the principal pathogenic mechanism. Consequently, this process results in the impairment of neuromuscular junction transmission, reducing the strength of voluntary muscle groups [1]. The role of the thymus gland in myasthenia gravis (MG) varies, depending on the specific form of MG and the existence of a thymoma. In situations of thymoma or early-onset myasthenia gravis associated with acetylcholine receptor antibodies, the thymus is involved in sickness beginning, frequently presenting indications of thymic hyperplasia. In contrast, the thymus does not seem to have a pathogenic function in MuSK MG. This variability shows the intricacy of MG and its varied processes, impacting the implications for pregnancy and fetal development [1,6].

Maternal IgG antibodies can navigate the placenta through Fc receptors, especially in the second and third trimesters of pregnancy. Consequently, pathogenic antibodies may access the fetus and disrupt neuromuscular transmission at the developing neuromuscular junction. The transplacental transfer may result in transient neonatal myasthenia gravisor, and in a few cases, fetal issues including diminished fetal movements, arthrogryposis multiplex congenita, or fetal akinesia due to disrupted neuromuscular interaction [7].

## 3. Newly Diagnosed Myasthenia Gravis with Pregnancy

Sex hormones, particularly estrogens and progesterone, may affect the etiology of MG, as evidenced by the higher prevalence of MG in females compared to males during reproductive years [6]. This hormonal effect may also play a part in the start of MG during pregnancy and during other times, such the postpartum period and breastfeeding, which could change how the disease progresses.

Throughout pregnancy, the modifications in the serum levels of sex hormones, as far as estrogen and progesterone are concerned, directly affect immune system function, and thus the progression of MG. The increase in progesterone during pregnancy leads to promotion of immunological tolerance by down-regulation of development of IL-17 produced by Th17 cells, as well as an increase in regulatory T-cells (T-regs), subsequently potentially suppressing inflammatory responses [8]. On the other hand, estrogens demonstrate a more fluctuating effect; at elevated levels, it might promote humoral immunity by activating T helper cell responses and facilitating the synthesis of anti-acetylcholine receptor (AChR) antibodies, thereby exacerbating MG symptoms. Estrogen additionally affects B cell functionality and the glycosylation profiles of immunoglobulin G (IgG), which undergo further modifications during pregnancy and may contribute to the immunopathology of the condition [8].

The prevalence of MG in women, particularly during reproductive years, highlights the crucial influence of sex hormones on disease presentation and severity. A wide range of immune cells, such as B and T lymphocytes, have receptors for steroid hormones, enabling direct regulation by estrogen and progesterone. Elevated estradiol during pregnancy might increase regulatory B cells and alter IgG glycosylation, thereby reducing inflammation; nevertheless, estrogen may also amplify the activity of pathogenic autoreactive B cells, hence exacerbating myasthenia gravis in certain patients. The postpartum phase, characterized by rapid hormonal fluctuations, is frequently associated with heightened exacerbations of myasthenia gravis, underscoring the significant role of sex hormones in the pathogenesis of myasthenia gravis throughout and after pregnancy [9].

A population-based cohort study done in Norway and the Netherlands looked back at 246 women who had myasthenia gravis (MG) between the ages of 15 and 45 and looked at the relative risk of getting MG before, during, and after pregnancy. The study indicated that there was no higher risk of MG starting during pregnancy. However, there was a much higher risk—almost five times higher—in the first six months after giving birth. The risk then returned to normal levels in the following six months. Fifteen percent of those with myasthenia gravis got it after becoming pregnant, usually during the postpartum period. After the first delivery, the risk was highest [10]. This study illustrates an inherent predisposition in specific women, whereby the initial postpartum period triggers the clinical manifestation of autoimmune myasthenia gravis. The study also shows links to other autoimmune diseases that have a higher risk of incidence after giving birth, like autoimmune thyroiditis and rheumatoid arthritis. People do not really know what creates this tendency. Some possible causes of this higher risk are a drop in alpha-fetoprotein levels after giving birth (which stops AChR antibodies from binding to acetylcholine receptors), an immune system rebound after delivery, exposure to fetal antigens, and the stress and physical changes that come with pregnancy and childbirth [10,11].

## 4. The Effect of Pregnancy on MG

Studies show that some women with MG do not find any change in their symptoms, but a large number do see either improvement or aggravation. During the first trimester and the first six months after giving birth, symptoms often get worse. Exacerbations are often mild to moderate, although myasthenic crises are seldom [11,12]. In contrast, enhancement often transpires during the second and third trimesters, presumably linked to pregnancy-induced immunosuppression and elevated alpha-fetoprotein levels [13]. In addition to the previously described hormonal and immunological aspects, the exacerbation of myasthenia gravis during pregnancy may be ascribed to respiratory muscle weakness, infections, specific drugs, and the stress associated with labor and delivery. The studies showed that there are no clear factors that can predict which MG patients will have flare-ups during pregnancy. Factors such as prior thymectomy, AChR antibody levels, or the period since the onset of MG seem to be inconsequential [14]. In general, more severe MG symptoms before pregnancy are likely to continue during the pregnancy. Also, how MG got worse in past pregnancies does not always indicate what will happen in future pregnancies. Moreover, investigations assessing the prevalence of clinical worsening in pregnant individuals with myasthenia gravis reveal a broad spectrum of effects throughout various trimesters and the postpartum period. Research shows that the rates of pregnancy worsening among women with myasthenia gravis (MG) are very different from one other. Reports show that these rates change a lot during pregnancy, from 10% to 90% [14]. This wide range shows how different pregnancies can affect MG in different ways. Research reveals that pregnancy is not a substantial risk factor for MG exacerbations, underscoring the relevance of non-pregnancy-related variables in the treatment of the condition both in the short and long term. The variables include the patient’s overall health, the severity and duration of myasthenia gravis, the presence of comorbidities, and potential genetic and environmental influences [14].

## 5. Women with Myasthenia Gravis Planning for Pregnancy

Myasthenia gravis (MG) is a disorder that affects women of reproductive age, often leading to the deferral or avoidance of pregnancy. It is advisable for women with myasthenia gravis to engage in preconception counseling with both an obstetrician and a neurologist, and they should be supported in their decision to pursue pregnancy if desired.

Immunosuppressive drugs are necessary for the efficient treatment of myasthenia gravis in most women of reproductive age. A fundamental issue is if these drugs hold any teratogenic potential. Methotrexate and mycophenolate mofetil are contraindicated prior to pregnancy. The use of methotrexate during pregnancy is connected with higher risk of neural tube defects including anencephaly as well as high rate of abortions. Consequently, the administration of methotrexate during gestation is forbidden, and women undergoing methotrexate treatment should apply contraceptive methods [15]. Methotrexate is characterized by the capacity to accumulate in cells. As a result, it should be stopped at least three months before trying to conceive. Furthermore, folic acid supplementation ought to be continued upon discontinuation, throughout preconception, and during pregnancy. Case reports have documented occurrences of low-dose methotrexate embryopathy, demonstrating that even modest doses may represent a risk [16]. Similarly, the use of cyclophosphamide during pregnancy is related to major congenital defects, and this medicine should be avoided in pregnant women.

As far as mycophenolate is concerned, its usage during pregnancy correlates with a heightened chance of first trimester miscarriage and an elevated likelihood of congenital abnormalities. A persistent pattern of congenital deformities has been found, including coloboma, cleft lip and palate, microphthalmia, and microtia atresia. Infrequently recorded abnormalities including anomalies of the heart, distal extremities, esophagus, vertebrae, kidneys, central nervous system, and diaphragm [13].

Prednisolone/prednisone and azathioprine are advised as primary therapies for myasthenia gravis, frequently utilized in conjunction. These medications are generally considered safe during pregnancy. In addition, fertility is not harmed by their use. The concentration of prednisolone and prednisone in fetal circulation is 10% or lower than that in the maternal circulation [17]. Prednisone and prednisolone should be administered only to women requiring this therapy for illness management. It is advisable to maintain the daily prednisone dosage at the lowest feasible level, preferably below 20 mg. Breastfeeding is advised with daily dosages under 20 mg. At elevated dosages, breastfeeding should be preferentially timed around corticosteroid administration.

Azathioprine is a mercaptopurine derivative that obstructs DNA replication and inhibits purine synthesis. Azathioprine is utilized for the management of certain autoimmune disorders and for individuals who have undergone organ or tissue transplantation. Current evidence does not indicate that azathioprine is a teratogen; however, some studies imply a heightened risk of preterm delivery and fetal growth limitation when the drug is administered during pregnancy [13].

Acetylcholine esterase inhibitors are utilized daily by nearly all patients with myasthenia gravis, as they enhance muscle strength specifically in this condition. Pyridostigmine is the preferred medication. This drug is considered safe during pregnancy and may be maintained without alteration while attempting to conceive and during pregnancy. The drug does not significantly cross the placenta. Some women opted to reduce or discontinue their pyridostigmine treatment during pregnancy. This may elucidate an exacerbation of myasthenia gravis during pregnancy [17].

Half of MG patients react to monoclonal IgG antibody rituximab. Its usage is not advised during or 12 months before pregnancy. Consensus-based treatment guidelines limit usage to 3 months before pregnancy or till conception. Rituximab has a 3-week elimination half-life and can deplete neonatal B cells. Eculizumab, an IgG antibody, reduces MG muscle weakness without affecting neonatal complement activity. Blocking FcRn receptors in children lowers IgG levels and may reduce the likelihood of neonatal myasthenia. Plasma exchange and intravenous immunoglobulin (IvIg) are effective therapies for MG exacerbations and MG crises, especially during pregnancy. Subcutaneous immunoglobulin is safe during pregnancy and beneficial in mild-to-moderate MG exacerbations [17].

Thymectomy is recommended for conceiving patients without MG thymectomy due to improved disease management and reduced risk of TNMG. Live-attenuated vaccinations should be administered after thorough evaluation. Pregnant patients should avoid infections, aspiration, or medication. Vaccination is recommended for all women, and a detailed record should be communicated to healthcare practitioners.

## 6. Surveillance of Pregnancy in Mothers with MG

### 6.1. First Trimester

A multidisciplinary team should provide prenatal care for expectant women with MG, with consultations adjusted to the patient’s clinical status. At least once per trimester, they should receive routine antenatal registration blood tests, AChR-Abs, and MuSK antibody levels. Folic acid supplementation should be maintained during the initial trimester [11]. Acetylcholinesterase inhibitors (pyridostigmine) are the initial treatment for symptomatic alleviation [18], with corticosteroids used as a secondary treatment if pyridostigmine doses are insufficient [19]. Nonsteroidal immunosuppressants may be used during pregnancy when corticosteroids are not well tolerated or when MG is refractory [19]. In severe crises, intravenous immunoglobulin (IVIg) or plasma exchange therapy may be considered as safe alternatives [11]. Vaccination against ultrasound scans at 11–13 weeks are beneficial for evaluating fetal anatomy and normal development. Non-invasive prenatal testing (NIPT) is permissible in pregnancies with MG [20]. High-risk pregnancies should be offered aspirin and patients should undergo a first trimester screening for preeclampsia [4].

### 6.2. Early Anomaly Scan: 16–18 Weeks

Maternal MG often results in fetal arthrogryposis multiplex congenita (AMC), a complex disorder that can be diagnosed during early pregnancy [21]. Prenatal ultrasound screening can be conducted as early as 16–18 weeks gestation. When many joints are impacted, the problem gets worse. The prognosis for infants with AMC is bleak because of its persistent and progressive characteristics. Early ultrasound discovery might help avert lung hypoplasia, which may lead to respiratory failure and mortality, and offer parents reproductive options, including the choice to terminate a pregnancy [22].

### 6.3. Routine Anomaly Scan: 20–24 Weeks

All women with myasthenia gravis (MG) should get regular anomaly scans to potentially identify significant fetal abnormalities at this gestational age. Despite this, there is no increased chance of giving birth to children with morphological or genetic defects. Growth, anatomy, fetal movements, and amniotic fluid volume assessment are used to assess fetal well-being. Invasive diagnostic procedures should be recommended if an ultrasound identifies a chromosomal or genetic anomaly [23]. Pregnant women diagnosed with myasthenia gravis should undergo a cervical evaluation during mid-pregnancy as an integral component of their preterm birth screening program.

### 6.4. Third Trimester Serial Growth Scans

Pregnant women possessing antibodies against fetal AChR frequently stay asymptomatic due to their incapacity to attach to the adult variant of AChR, commonly located in maternal muscles. Symptomatic or asymptomatic cases should be evaluated by ultrasound scans to assess fetal joint morphology and amniotic fluid content. Diagnosis of polyhydramnios in a pregnancy with a fetus with fixed joints raises suspicion of arthrogryposis multiplex congenita (AMC) [22]. Ultrasound scans may be necessary every 2–4 weeks for women with myasthenia gravis (MG) to evaluate fetal development, well-being, movements, and amniotic fluid volume, depending on the clinical conditions. The standard prenatal vaccination protocol should be adhered to for all patients.

### 6.5. Intrapartum Care and Deliver Considerations

Pregnancy risk should be examined considering maternal and fetal problems, not simply myasthenia gravis. For all pregnancies involving MG women, intrapartum observation is advised. Pregnant women with myasthenia gravis can safely give birth vaginally, and this should be encouraged. Cesarean delivery should be conducted purely for obstetric concerns, as the technique is usually associated with the worsening of myasthenia gravis and may induce a myasthenic crisis. The uterus, constituting smooth muscle, is unaffected by the disease process in myasthenia gravis, and its contractility stays intact. Thus, magnesium does not affect the initial phase of labor. Nonetheless, the second stage of labor demands the engagement of striated muscle, which may lead to patient fatigue, sometimes necessitating the use of forceps or vacuum extraction [13].

Epidural analgesia should be employed to alleviate pain during childbirth. Narcotic and neuromuscular blocking drugs should be avoided. Local anesthetic drugs should be avoided when feasible, as they may inhibit neuromuscular transmission. Nondepolarizing neuromuscular blocking agents should be avoided. Sedatives and opioids should be avoided, as they may induce respiratory depression. If these medications are necessary, women should be closely monitored for respiratory functions. Women on chronic low-dose steroids may get a stress dose of hydrocortisone during the intrapartum period. Cholinesterase inhibitors may be administered parenterally if necessary, with neostigmine being the preferred option. In women with preeclampsia and eclampsia, the use of magnesium sulfate should be avoided as it may disrupt neuromuscular transmission by inhibiting the production of acetylcholine [13].

### 6.6. Postpartum Care and Neonatal Monitoring

Myasthenia gravis (MG) during pregnancy is unpredictable but may worsen postpartum. Postnatal consultation with a multidisciplinary team is crucial for adjustment of treatment doses. Breastfeeding is contraindicated for newborns with TNMG due to AChR-Abs transfer. Anticholinesterase inhibitors like pyridostigmine and neostigmine are safe for breastfeeding, while mothers receiving azathioprine or mycophenolate should avoid breastfeeding.

Mothers with myasthenia gravis have a 10–20% risk of having a child affected by transient neonatal myasthenia gravis. The risk is elevated in women experiencing exacerbations of myasthenic symptoms, particularly in those who are hesitant to undergo treatment or have not had a thymectomy. Around 70% of infants with this condition show symptoms within the first day of life, and in rare cases, they last up to 4–7 days. During the first three weeks after delivery, maternal antibodies are removed from neonatal circulation. Neonatal myasthenia gravis symptoms, such as difficulty sucking, palpebral ptosis, facial paresis, and/or generalized hypotonia, are usually mild to moderate. Respiratory support and tube feeding are infrequently necessary. To determine whether the neonate’s swallowing or breathing muscles are involved, close observation is crucial. This syndrome is temporary and often remits spontaneously; however, in some situations, a high-risk nursery may be necessary [24].

## 7. Role of Autoantibodies in the Detection of Myasthenia Gravis

The discrepancy of MG affects the prognosis of disease development, as antibody levels often show no correlation with clinical severity or response to therapy. Although antibodies are significant for diagnosis, more accurate biomarkers are essential to enhance risk stratification, inform therapeutic decisions, and forecast disease progression, especially during pregnancy, when early detection is vital. Effective biomarkers must have high specificity and sensitivity for patient identification.

The diagnosis of MG often starts with the laboratory identification of autoantibodies targeted against the five subunits of the muscle acetylcholine receptor (AChR), predominantly of the IgG1 or IgG3 subclasses, utilizing a radioimmunoprecipitation assay (RIPA) [25]. While total AChR antibody titers do not consistently reflect the severity of MG, there is some data indicating that the IgG1 subtype titer may correlate with a more severe clinical presentation. AChR antibodies can be identified up to two years prior to the development of symptoms, considering them significant for early diagnosis [26].

MuSK antibodies, classified as IgG4, are identified in around 6% of all MG patients, 40% of acetylcholine receptor antibody-negative cases, and up to 12.5% of acetylcholine receptor antibody-positive cases [25]. These antibodies specifically target the extracellular domains and cysteine-rich areas of MuSK, thereby disrupting critical interactions that facilitate AChR clustering at the neuromuscular junction.

LRP4 antibodies, predominantly of the IgG1 subclass, participate in complement activation and contribute to the development of MG by disrupting agrin-mediated clustering of acetylcholine receptors [27,28].

Titin and ryanodine receptor (RyR) antibodies serve as predictive indicators for thymoma in early-onset MG, associated with heightened disease severity and thymoma prevalence. Agrin antibodies, although infrequent in MG and nonexistent in other neurological illnesses and healthy individuals, have potential as highly specific biomarkers for MG, especially as they are also present in patients with antibodies against AChR, MuSK, or LRP4 [29].

In summary, although several antibodies fulfil diagnostic and prognostic functions in MG, the ongoing search for accurate and disease-specific biomarkers remains essential to promoting earlier detection, customized treatment, and outcome prognosis in patients with MG.

## 8. Non-Coding RNAs and Their Role in the Early Detection and Prognosis of MG

Myasthenia gravis is an autoimmune condition characterized by multifactorial inheritance. Epigenetic variables, including non-coding RNAs, have been identified as influencing the etiology of this condition, perhaps establishing a connection between environmental and genetic factors. MicroRNAs (miRNAs) are diminutive, non-coding endogenous RNA molecules that function at the post-transcriptional level, regulating gene expression through several methods [30]. Recently, researchers assessed the potential of miRNAs as significant biomarkers for illness detection, complication identification, and predicting therapeutic responses. Furthermore, miRNAs were identified in the extracellular space, blood plasma, serum, amniotic fluid, cerebral fluid, peritoneal/pleural fluids, breast milk, urine, and tears, referred to as circulating miRNAs [31,32]. Long non-coding RNAs (lncRNAs) are additional circulating RNAs comprising over 200 nucleotides. Long non-coding RNAs (lncRNAs) can influence immune cell phenotypes, change the balance between Th17 and T-reg cells, and regulate the release of pro-inflammatory cytokines [33]. MiRNAs have been studied more extensively in relation to MG than lncRNAs. The benefits of using circulating RNAs include their resilience to fluctuations in temperature and pH due to membrane encapsulation. The straightforward identification of miRNAs in bodily fluids serves as an effective instrument for the early diagnosis and subtyping of MG. Furthermore, the therapeutic response in myasthenia gravis patients may be assessed by the use of circulating RNAs [34].

Table 1 contains some of the most studied miRNAs in the context of MG course, progression and response to treatment interventions.

## 9. Conclusions

With two peaks in incidence (in the third decade and after the sixth decade), myasthenia gravis has a bimodal age distribution. In the younger age group, it primarily affects females. A contrast is drawn between a generalized form and an ocular form, with the latter exhibiting a more favorable prognosis. An elevated related risk of preterm delivery is suggested; however, other pregnancy problems do not seem to be heightened. The progression of myasthenia gravis during pregnancy differs significantly across individuals. Although the disease remains constant in numerous pregnant women, it may also deteriorate, and in a minority of cases, even ameliorate. Deterioration manifests throughout the first or second trimester and/or postnatally. Myasthenic crises during pregnancy must be handled in accordance with established treatment protocols, such as intravenous immunoglobulins or plasmapheresis, and addressed as an emergency by a multidisciplinary team. For the identification, diagnosis, and differential diagnosis of maternal illness-specific symptoms and consequences, it is imperative to guarantee tight collaboration among specialists in neurology, fetal and maternal medicine, and neonatology in instances of active disease. In the overall management of myasthenia gravis, the minimal effective steroid dosage should be chosen. The treatment of magnesium for preeclampsia in afflicted pregnant patients may result in significant deterioration.

Thymectomy, if intended, should be performed prior to pregnancy. Preconception counseling needs to be provided to all women with myasthenia gravis. All women ought to get regular examinations during the prenatal phase. A comprehensive list of all medications that are known to exacerbate myasthenia gravis should be provided to all women diagnosed with the condition.

The management of myasthenia gravis during gestation must be tailored to the individual patient. Anticholinesterase medications may be adequate for women with modest isolated ocular weakness, but immunosuppression is frequently required for more severe myasthenia gravis conditions. Steroids are the preferred immunosuppressant medication. These should be administered at the minimum effective dosage. In individuals who are intolerant or resistant to steroids, azathioprine or cyclosporine may serve as alternative immunosuppressants.

Vaginal delivery should be pursued in all women with myasthenia gravis. Cesarean sections should be conducted solely for obstetric grounds. The initial phase of labor is often not influenced by myasthenia gravis. If the mother is tired, the second stage of MG may need to be shortened. During labor, sedatives, magnesium, and nondepolarizing muscle relaxants must be avoided. If a vaginal birth is expected, regional anesthetics may be used. All neonates given to women with myasthenia gravis should be thoroughly watched for a minimum of 72 h due to the potential of transitory neonatal MG, which occurs in 10–20% of newborns born to myasthenic mothers.

The use of several biomarkers in the diagnosis, treatment, and prognosis of MG during pregnancy may help to identify MG from its differential diagnoses and address the management challenges that these patients endure. Despite various research targeted at identifying new biomarkers, considerable challenges remain in characterizing the profile of MG biomarkers. This publication gives a brief overview of immunological biomarkers, RNAs, and other new biomarkers in myasthenia gravis (MG). More research is needed to determine the specific role of these biomarkers in the pathology of myasthenia gravis during pregnancy, to evaluate biomarker profiles across different subgroups of the condition, and to assess changes in biomarkers following the implementation of treatment protocols.

MiRNAs and lncRNAs work together to regulate MG pathogenesis, progression, and immunosuppressive therapy efficacy. As a result, these transcripts may serve as indications for predicting specific qualities. High-throughput investigations are required to uncover variables impacting lncRNAs and miRNAs both upstream and downstream, given the complex involvement of genetic determinants in MG development. This project will identify novel transcription factor/lncRNA/miRNA/mRNA axes that could be implicated in the etiology of MG.

## Figures and Tables

**Table 1:** Most studied microRNAs in association with myasthenia gravis.

miRNA	MG Subtype	Up- or Down-Regulated/Levels in Serum After Therapeutic Interventions	Reference
miR-150-5p	AChR + MG	Linked to B cell activation autoantibody production. Upregulated expression/Upregulated levels in serum. Downregulated after therapeutic interventions like thymectomy or immunosuppressive therapies.	[35,36,37]
miR-21-5p	AChR + MG	Upregulated in serum before intervention. Downregulated after immunosuppressive treatment.	[32,37]
miR-30e-5p	AChR + MG	Downregulated in serum of EOMG patients. Upregulated in serum of LOMG patients. Levels correlate with disease course but not with severity.	[32]
miR-146a	AChR + MG	Modulates innate immunity. Upregulated in serum of patients with MG. Upregulated in serum of patients treated with corticosteroids.	[38,39]

## Data Availability

No new data were created or analyzed in this study. Data sharing is not applicable to this article.
